# Cannabis (THC) Aggravates the Deleterious Effects of Alcohol (EtOH) on Skeletal Muscles’ Mitochondrial Respiration: Modulation by Age and Metabolic Phenotypes

**DOI:** 10.3390/biology13121080

**Published:** 2024-12-21

**Authors:** Anne-Laure Charles, Margherita Giannini, Alain Meyer, Anne Charloux, Samy Talha, Thomas Vogel, Jean-Sébastien Raul, Valérie Wolff, Bernard Geny

**Affiliations:** 1 UR 3072, “Mitochondria, Oxidative Stress and Muscle Plasticity”, Biomedicine Research Center of Strasbourg (CRBS), Faculty of Medicine, University of Strasbourg, 67000 Strasbourg, France; anne.laure.charles@unistra.fr (A.-L.C.); margherita.giannini@chru-strasbourg.fr (M.G.); alain.meyer1@chru-strasbourg.fr (A.M.); anne.charloux@chru-strasbourg.fr (A.C.); samy.talha@chru-strasbourg.fr (S.T.); thomas.vogel@chru-strasbourg.fr (T.V.); valerie.wolff@chru-strasbourg.fr (V.W.); 2 Physiology and Functional Explorations Department, University Hospital of Strasbourg, 67000 Strasbourg, France; 3 Geriatrics Department, University Hospital of Strasbourg, 67200 Strasbourg, France; 4 Toxicology Laboratory, Institute of Legal Medicine, Faculty of Medicine, University of Strasbourg, 67000 Strasbourg, France; js.raul@unistra.fr; 5 Neuro-Vascular Department, University Hospital of Strasbourg, 67200 Strasbourg, France

**Keywords:** skeletal muscle, metabolic phenotype, glycolytic, oxidative, aging, mitochondria, tetrahydrocannabinoid, cannabis, marijuana, THC, alcohol, ethanol, EtOH

## Abstract

The anti-inflammatory and analgesic properties of cannabis might be useful to treat muscle diseases, including those linked or not to alcohol. Nevertheless, delta 9 tetrahydrocannabinol (THC) and ethanol (EtOH), often used concomitantly, can have deleterious effects on cardiac mitochondria. We therefore determined whether EtOH, alone and associated with THC, impairs skeletal muscle mitochondrial respiration. Further, we investigated potential modulation by metabolic phenotype and age by analyzing predominantly glycolytic gastrocnemius and oxidative soleus muscles in young and middle-aged rats (12 and 49 weeks). Considering the gastrocnemius, EtOH impaired mitochondrial respiration in a similar manner in young- and middle-aged muscles (−34.97 ± 2.97% vs. −37.50 ± 6.03% at 2.1 × 10^−5^ M; *p* < 0.05). Interestingly, concomitant THC aggravated EtOH-related mitochondrial impairment in young gastrocnemius (−49.92 ± 1.69%, vs. −34.97 ± 2.97 *p* < 0.05). Concerning the soleus, EtOH alone mainly decreased young muscle mitochondrial respiration (−42.39 ± 2.42% vs. −17.09 ± 7.61% at 2.1 × 10^−5^ M, *p* < 0.001, at 12 and 49 weeks). The soleus was less impaired at 12 weeks by THC and EtOH association than the gastrocnemius (−49.92 ±1.69 vs. −27.22 ± 8.96% in gastrocnemius and soleus, respectively, *p* < 0.05). In conclusion, EtOH, alone and associated with THC, significantly impairs skeletal muscle mitochondrial respiration and THC aggravates EtOH-induced effects on young glycolytic muscle. Age and metabolic phenotypes modulate these deleterious effects, with the glycolytic muscles of young rats being more prone to impairments than oxidative muscles.

## Simple Summary

Cannabis (THC) and ethanol (EtOH) are widely used for their anti-inflammatory and analgesic properties. Whether both drugs have deleterious effects on skeletal muscle needs further investigations, particularly looking at mitochondria, the energy producers of the cells. We determined the effects of EtOH, alone and associated with THC, on skeletal muscle mitochondrial respiration, on predominantly glycolytic gastrocnemius muscles (less mitochondria) and oxidative soleus (many mitochondria) muscles in young and middle-aged rats (12 and 49 weeks). Considering the gastrocnemius, EtOH impaired mitochondrial respiration in a similar manner in young- and middle-aged muscles (−34.97 ± 2.97% vs. −37.50 ± 6.03%). Interestingly, concomitant THC aggravated EtOH-related mitochondrial impairment in young gastrocnemius muscles (−49.92 ± 1.69%, vs. −34.97 ± 2.97). Concerning the soleus, EtOH alone mainly decreased young muscle mitochondrial respiration (−42.39 ± 2.42% vs. −17.09 ± 7.61%, at 12 and 49 weeks). The soleus was less impaired at 12 weeks by THC and EtOH association than the gastrocnemius. In conclusion, EtOH, alone and associated with THC, significantly impairs skeletal muscle mitochondrial respiration and THC aggravates EtOH-induced alterations in young glycolytic muscle. Caution is therefore warranted if using THC or EtOH alone, and even more caution is needed if both drugs are concomitantly used.

## 1. Introduction

After alcohol and tobacco, cannabis is the third most used drug in the world. Cannabis has been authorized in many countries either for recreative or for therapeutic purposes. Indeed, cannabidiol has been shown to be useful to treat patients suffering from some chronic diseases and cannabis therapeutic indications are growing in number [1,2,3,4]. The anti-inflammatory and analgesic properties of cannabis might be particularly useful in pathologies involving skeletal muscles such as neuromuscular disorders, cancer-related pain, etc. [3,5,6]. Indeed, muscle impairments are often involved in patients’ reduced quality of life through muscular pain and reduced strength. 

On the other hand, cannabis and its main psychoactive component Delta 9 tetrahydrocannabinol (THC) can induce severe side effects. Although the primary issues of THC and ethanol (EtOH) use are addictions, THC also favors cardiovascular alterations. Thus, THC was associated with myocardial infarction whose prognosis appeared worse when related to cannabis [7,8,9] and with a high frequency of intracranial arterial stenosis in ischaemic stroke in young patients [10]. Indirect mechanisms participate in these deleterious effects. Besides activation of the sympathetic system and pro-coagulation cascade, arterial narrowing leading to severe central and peripheral ischemia–reperfusion consequences has been reported [9,11,12,13,14,15,16].

Additionally, THC also directly impairs mitochondrial respiration in several tissues, including the brain and the heart [17,18]. Cannabis-induced learning/memory deficits in rats have been associated with reduced brain mitochondrial respiration and a reduced threshold for mitochondrial permeability transition and calcium uptake [19].

We recently reported cumulative deleterious effects of THC and EtOH on cardiac mitochondrial respiration, raising the issue that other striated muscles might be sensitive to both drugs [20]. Accordingly, cannabinoid type 1 receptors were found in skeletal muscles and their ablation increased muscle mass and improved physical performance, together with increased oxidative capacity and shifts from Type II to Type I fibers [21,22]. Importantly, mitochondrial content also appeared to predict the mortality of patients with peripheral arterial disease [23]. Despite this significance, limited data are available concerning the effects of THC on skeletal muscles’ mitochondrial function.

From this perspective, the metabolic phenotype of striated muscle shows potential. Soleus and gastrocnemius muscles are predominantly oxidative and predominantly glycolytic, respectively, and interestingly, the soleus is less sensitive to ischemia–reperfusion-related damage than glycolytic gastrocnemius muscles [24,25,26].

Additionally, aging generally enhances diseases severity and drug-related deleterious effects. This is true during lower-limb ischemia–reperfusion and recently, aging has been shown to enhance the cannabis-related toxic effects on cardiac mitochondria [20,27,28,29]. There is a relationship between age and cannabinoid receptors in skeletal muscles. Aged skeletal muscles concurred with increased CB1R mRNA abundance and CB1 receptor blockade counteracts age-induced insulin resistance and metabolic dysfunction [30]. Further, Dalle et al. recently observed in human muscles that the expression of cannabinoid receptor CB_1_ is higher in old muscle compared to young muscle [31]. On the other hand, young age was likely to be associated with cannabis-related stroke [10]. The potential toxic effect of THC on skeletal muscles might therefore vary depending on both age and muscle characteristics, supporting the need to investigate not only young and middle-aged muscles but also muscles with different metabolic phenotypes.

Concerning alcohol, Lewitt et al. reported recently that short-term in vitro ethanol (EtOH) exposure can decrease myoblast mitochondrial membrane potential, together with a small reduction in mitochondrial ATP production [32]. EtOH has also been shown to acutely impair skeletal muscle mitochondrial respiration of heart-transplanted patients [33]. Thus, ethanol is a myotoxic compound able to induce dilated cardiomyopathy and skeletal muscle alterations. Accordingly, cytochrome c oxidase activity and mitochondrial volume were impaired in patients misusing alcohol and EtOH decreased mitochondrial respiration in myotubes [34,35,36,37]. Thus, as previously observed in the liver, alcohol-mediated changes in mitochondrial morphology, biogenesis and dynamics (i.e., mitochondrial quality control mechanisms crucial for optimal mitochondrial function) associated with increased oxidative stress likely participate in muscle impairment [38,39]. On the other hand, reports have suggested that EtOH might not significantly impair skeletal muscle mitochondrial functions [40,41]. Thus, inconsistent data have been reported regarding the effects of EtOH on skeletal muscles’ mitochondria, depending on the diverse EtOH exposures (for a review, see [38,42]). Nevertheless, as proposed by Dileao et al., since EtOH consumption largely varies in humans, each study provides interesting information that enhances our comprehension of EtOH-induced effects on skeletal muscle mitochondrial function [42]. 

Furthermore, people frequently consume cannabis and alcohol concomitantly. Therefore, it would be interesting to determine whether such an association would result in enhanced effects on skeletal muscles. Indeed, previous reports demonstrated that THC together with EtOH resulted in greater cardiovascular and cognitive impairments compared to each drug when used alone [43,44]. Alcohol and cannabis use may also contribute to synergistic priming of more severe inflammation within the lung in the setting of pulmonary infection [45]. 

To the best of our knowledge, limited data are available concerning the potential direct toxic effects of cannabis or ethanol alone and in association on skeletal muscles’ mitochondria.

The aim of this study was therefore to determine for the first time the effects of EtOH, alone and associated with THC, on skeletal muscle mitochondrial respiration. Further, we investigated the potential modulation by metabolic phenotype and age, analyzing predominantly glycolytic gastrocnemius and oxidative soleus muscles in young and middle-aged rats.

## 2. Materials and Methods

### 2.1. Study Design

Experiments were performed on young 12-week-old (n = 4; 4–10 runs) and middle-aged 49-week-old (n = 3; 5–11 runs) male Wistar rats (Janvier, Le Genest-St-Isle, France). Power calculation using G*power software 3.1.9.7 (Heinrich-Heine-University, Düsseldorf, Deutschland) was based on a comparison of means between two groups of the same size, a power of 80% and an α risk of 5%. According to previous data obtained on cardiac mitochondria at 12 weeks [20], the sample size should be a minimum of 4 per group. For older rats, the sample size should be a minimum of 3 per group. Following the good practice rule aiming to reduce the number of animals involved in research, we nevertheless fulfilled the requirements for statistically rigorous results. Animals were housed at 22 ± 2 °C, with a 12 h light–dark cycle, water, and food ad libitum, and with an enriched environment. As previously reported, middle aged refers to adult rats, with a corresponding human age of about 30 years [46].

This investigation was carried out in accordance with “the principles of laboratory animal care” and respected the Guidelines of the European Union (86/609/EU) and the Committee for the Care and Use of Laboratory Animals (Cremeas, Strasbourg, France, decree 2013-118, article. R. 214-89). More precisely, the study was performed according to the article R214-89, modified by decree 2020-274, 17 March 2020—art. 1: “The killing of animals for the sole purpose of using their organs or tissues, in accordance with a method defined by joint order of the Minister of Agriculture and the Minister of Research, is not considered an experimental procedure”.

Rats were anesthetized with 3% isoflurane in an induction chamber (Minerve, Esternay, France), and then decapitated. The skeletal muscles were excised and immediately placed in an ice-cold isolation buffer. To investigate the dose–response effect of THC on mitochondrial respiration, synthetic THC (C_21_H_30_O_2_ diluted 25 mg/mL in ethanol, C_2_H_5_OH, Sigma Aldrich, St. Louis, MO, USA) was injected in the respiration chamber at the following concentrations (1 × 10^−5^, 5 × 10^−5^, 0.1 × 10^−3^, 0.15 × 10^−3^, and 0.2 × 10^−3^ M), based on previous data [20]. Muscles were exposed to THC for 20 min, with a concentration increase every 4 min. To determine the specific amount of ethanol (the solvent), it was also used alone at concentrations of 0.1 × 10^−5^, 0.5 × 10^−5^, 1.1 × 10^−5^, 1.6 × 10^−5^ and 2.1 × 10^−5^ M during five successive injections, corresponding to the dose used for THC solubilization.

### 2.2. Mitochondrial Respiration

We studied the mitochondrial respiration of skinned fibers rather than isolated mitochondria to preserve the environment and the integrity of mitochondria. Indeed, a comparison of rates of respiration of isolated human skeletal muscle mitochondria and saponin-skinned muscle fibers showed that these fibers can replace isolated mitochondria [47,48,49,50]. The two muscles studied were predominantly glycolytic *gastrocnemius* and oxidative *soleus* muscles. Fibers were separated using a magnifying glass and permeabilized in solution S (CaK_2_EGTA 2.77 mM, K_2_EGTA 7.23 mM, Na_2_ATP 6.04 mM, MgCl_2_ 6.56 mM, taurine 20 mM, sodium phosphocreatine 12.3 mM, imidazole 20 mM, dithiothreitol 0.5 mM, K-methane sulfonate 50 mM, pH 7.0) containing 50 μg/mL saponin for 30 min at 4 °C, under gentle shaking. Permeabilized fibers were then washed for 10 min in solution S to remove saponin and placed in a bath with the respiratory solution (CaK_2_EGTA: 2.77 mM, K_2_EGTA: 7.23 mM, MgCl_2:_ 6.56 mM, taurine: 20 mM, K_2_HPO_4_: 3 mmol/L, imidazole: 20 mM, dithiothreitol: 0.5 mM, K-methane sulfonate: 50 mM, and 2 mg/mL: bovine serum albumine, at pH 7.0), for two 5 min periods, to remove all phosphates.

Oxygen consumption was measured by using a Clark-type electrode in an oxygraphic cell (Strathkelvin Instruments, Glasgow, Scotland), as previously described [26,50,51]. Mitochondrial respiration corresponding to non-phosphorylating respiratory activity through complex I was explored via injection of glutamate (5 mM) and malate (2 mM) and oxidative phosphorylation activity (OXPHOS CI) was determined after ADP injection (2 mM), which activates ATP synthase at 22.1 °C under continuous stirring in the presence of a saturating amount of adenosine diphosphate as a phosphate acceptor. Then, five increasing doses of EtOH alone or associated with THC were injected, as described above. After the experiment, fibers were harvested and dried for 15 min at 150 °C, and respiration rates were expressed as μmol O_2_/min/g dry weight.

### 2.3. Statistical Analysis

All data were expressed as mean ± standard error of the mean (SEM). The statistical analyses were performed using Prism software (GraphPadPrism 8.4.3, GraphPad Software, San Diego, CA, USA). After checking normality with the Shapiro–Wilk test, one-way ANOVA was performed with the Dunnett post hoc test to analyze the parameters’ evolution following THC or vehicle exposures. For the samples, following a normality test, Student’s two-tailed *t*-test was used for group comparisons, and for other comparisons, a Mann–Whitney test was performed. A *p*-value < 0.05 was considered statistically significant.

## 3. Results

### 3.1. Baseline Mitochondrial Respiration in Glycolytic, Oxidative, Young, and Middle-Aged Skeletal Muscles

#### 3.1.1. Enhanced Mitochondrial Respiration in Young Oxidative Muscle as Compared to Young Glycolytic Muscle

**Figure 1:**
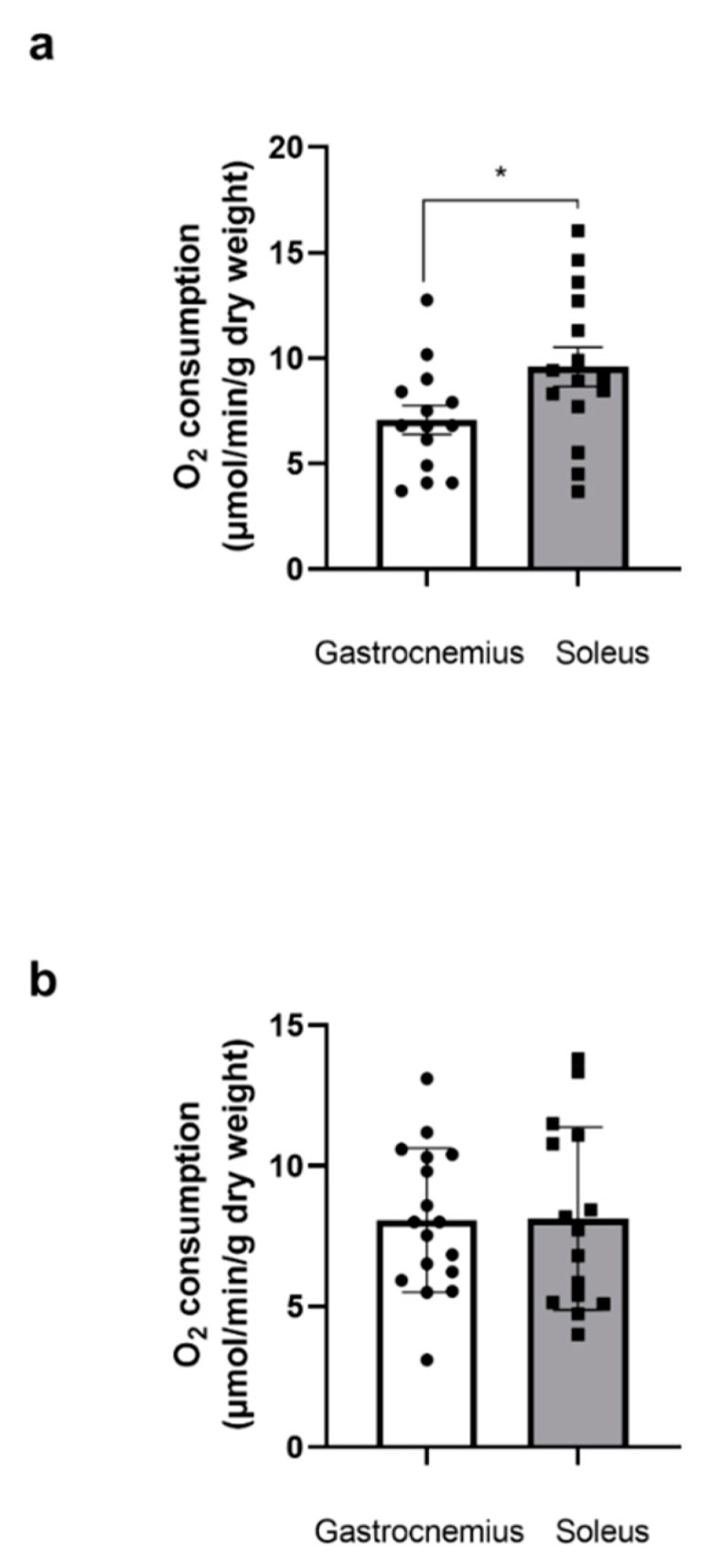
Baseline gastrocnemius and soleus muscles’ mitochondrial respiration before THC or EtOH addition at 12 weeks (**a**) or 49 weeks (**b**). Mitochondrial respiration before THC or EtOH addition, corresponding to the 100% values shown in other figures. * *p* < 0.05. n = 4 (14–15 runs) for 12 weeks; n = 3 (n = 15–17 runs) for 49 weeks.

Before THC or EtOH addition, mitochondrial respiration was significantly higher in the soleus at 12 weeks compared to the gastrocnemius at the same age (9.58 ± 0.93 vs. 7.07 ± 0.68 μmol O_2_/min/g dry weight, *p* < 0.05, Figure 1a). 

#### 3.1.2. Similar Mitochondrial Respiration in Middle-Aged Glycolytic and Oxidative Muscles

At 49 weeks, there was no difference in mitochondrial respiration between the two muscles (8.07 ± 0.62, and 8.13 ± 0.84 μmol O_2_/min/g dry weight) for the gastrocnemius and the soleus, respectively (Figure 1b).

### 3.2. Effect of Ethanol Alone (EtOH) or Associated with Tetrahydrocanabinoid (THC/EtOH) on Gastrocnemius Mitochondrial Respiration

#### 3.2.1. Similar EtOH Impairment in Young- and Middle-Aged Gastrocnemius

In the 12-week-old rats, EtOH dose-dependently decreased the mitochondrial respiration (−34.97 ± 2.97% at 2.1 × 10^−5^ M, *p* < 0.05).

**Figure 2:**
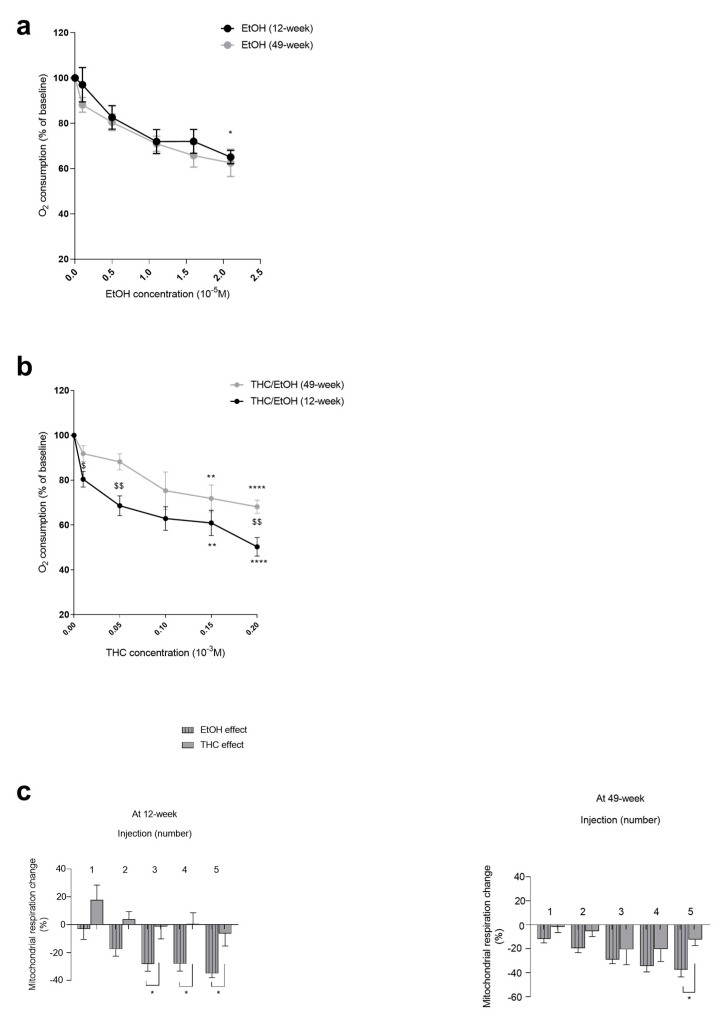
Dose–response effect of EtOH alone and of EtOH with THC on gastrocnemius mitochondrial respiration in 12-week-old and 49-week-old rats. (**a**) Effect of EtOH alone. (**b**) Effect of concomitant EtOH and THC. (**c**) Effect of THC and EtOH contributions to mitochondrial respiration in the gastrocnemius at 12 weeks or 49 weeks. The injection numbers correspond to the concentrations of EtOH (from 0.1 × 10^−5^ to 2.1 × 10^−5^ M) or THC/EtOH (from 1 × 10^−5^, to 0.2 × 10^−3^ M). Values are means ± SEM. * *p* < 0.05, ** *p* < 0.01, **** *p* < 0.0001 vs. baseline. Comparisons between groups $ *p* < 0.05, $$ < *p* < 0.01. EtOH: ethanol. THC: tetrahydrocannabinoid. n = 4 for 12 weeks; n = 3 for 49 weeks.

In the 49-week-old rats, the EtOH-related decrease in mitochondrial respiration was similar (−37.50 ± 6.03% at 2.1 × 10^−5^ M), (Figure 2a).

#### 3.2.2. Concomitant THC Aggravated EtOH-Related Mitochondrial Impairment in Young Gastrocnemius

Thus, at 12 weeks, the EtOH, associated with THC, dose-dependently decreased the mitochondrial respiration by −49.92 ± 1.69% at 0.2 × 10^−3^ M (*p* < 0.0001). Thus, THC in addition to EtOH further aggravated the gastrocnemius mitochondrial respiration impairment (−34.97 ± 2.97 and −49.92 ± 1.69%, *p* < 0.05), (Figure 2b). 

At 49 weeks, THC/EtOH also decreased gastrocnemius mitochondrial respiration (−31.79 ± 2.36% at 0.2 × 10^−3^ M, *p* < 0.0001 (Figure 2b)), but no significant difference was observed when comparing to EtOH alone.

Based on these data, we aimed to assess THC and EtOH relative effects on mitochondrial respiration and, as previously reported [20], we calculated the percent (%) changes from baseline related to THC alone obtained by subtracting the EtOH alone effect from the global effect of THC associated with EtOH.

In 12-week-old gastrocnemius, the decrease in mitochondrial respiration was mainly due to EtOH alone. The difference was −34.97 ± 2.97 vs. −6.31 ± 8.96%, with *p* < 0.05 for EtOH and THC, respectively, at the higher dose (Figure 2c).

In 49-week-old rats, the decrease in mitochondrial respiration was also mainly due to ethanol. Thus, the maximal decrease in oxygen consumption was −37.5 ± 6.03% due to ethanol alone and −12.35 ± 4.97% due to THC, where *p* < 0.05 (Figure 2c). 

### 3.3. Effect of Ethanol Alone (EtOH) or Ethanol Associated with THC (THC/EtOH) on Soleus Mitochondrial Respiration

#### 3.3.1. EtOH Mainly Decreased Young Soleus Mitochondrial Respiration

**Figure 3:**
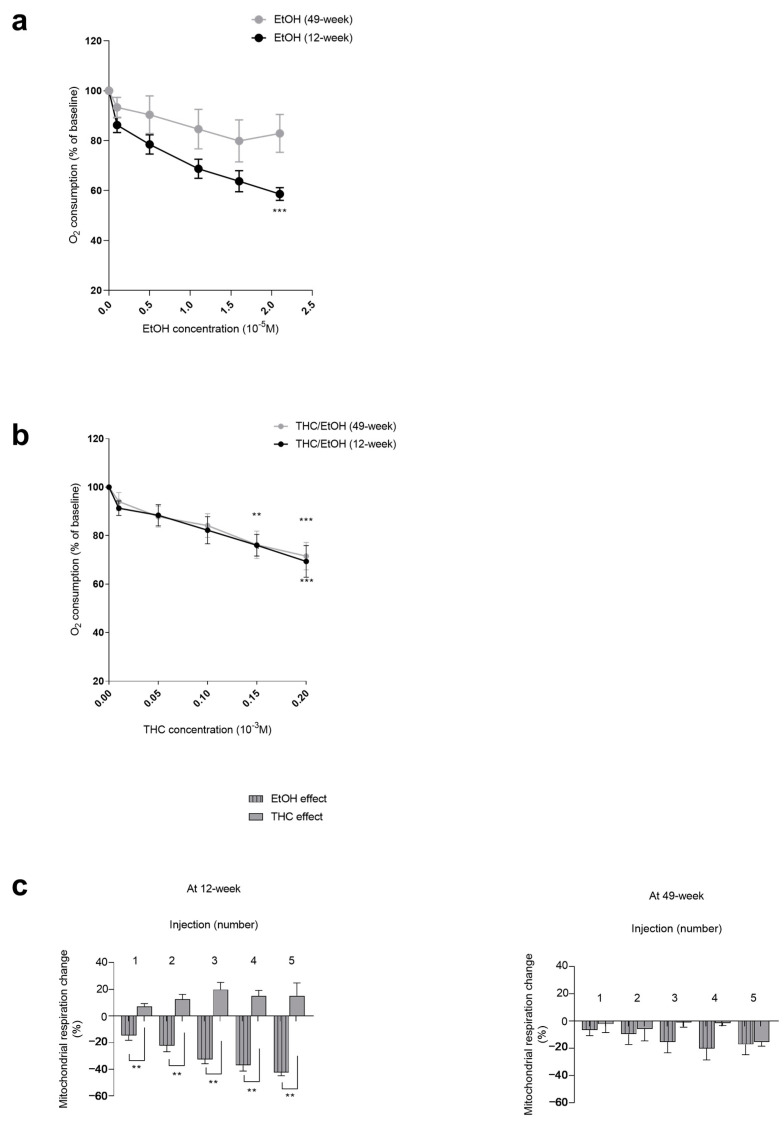
Dose–response effect of EtOH alone and a combination of EtOH and THC on soleus mitochondrial respiration in 12-week-old and 49-week-old rats. (**a**) Effect of EtOH alone. (**b**) Effect of concomitant EtOH and THC. (**c**) Effect of THC and EtOH contributions to mitochondrial respiration in the soleus at 12 weeks or 49 weeks. The injection numbers correspond to the concentrations of EtOH (from 0.1 × 10^−5^ to 2.1 × 10^−5^ M) or THC/EtOH (from 1 × 10^−5^, to 0.2 × 10^−3^ M). Values are means ± SEM. ** *p* < 0.01, *** *p* < 0.001 vs. baseline of each group. EtOH: ethanol. THC: tetrahydrocannabinoid. n = 4 for 12 weeks; n = 3 for 49 weeks.

EtOH significantly decreased the soleus mitochondrial respiration in 12-week-old muscles (−42.39 ± 2.42% at 2.1 × 10^−5^ M, *p* < 0.001). The soleus mitochondrial respiration decreases failed to reach statistical significance in 49-week-old muscles (−17.09 ± 7.61% at 2.1 × 10^−5^ M) (Figure 3a). 

#### 3.3.2. Concomitant THC and EtOH Similarly Impaired Middle-Aged and Young Soleus Muscles

Thus, the soleus mitochondrial respiration decreased in a similar for both ages (−27.22 ± 8.96, where *p* < 0.001, and −28.32 ± 7.32%, where *p* < 0.001, at 0.2 × 10^−3^ M for middle-aged and young muscles, respectively) (Figure 3b).

We aimed to assess THC and EtOH relative effects on mitochondrial respiration at 12 and 49 weeks.

In 12-week-old soleus, the decrease in mitochondrial respiration was mainly due to EtOH alone, as compared to THC. The difference was significant (−42.39 ± 2.42 vs. +15.17 ± 9.64%, *p* < 0.01) at the higher dose (Figure 3c).

In 49-week-old rats, the decrease in mitochondrial respiration tended also to be mainly due to ethanol, but the difference failed to reach statistical significance. Thus, the maximal decrease in oxygen consumption was −17.09 ± 7.61% for ethanol alone and −15.34 ± 3.12% for THC (Figure 3c).

### 3.4. Age and Metabolic Phenotype Modulates EtOH Associated with THC Deleterious Effects on Skeletal Muscle Mitochondrial Respiration

**Figure 4:**
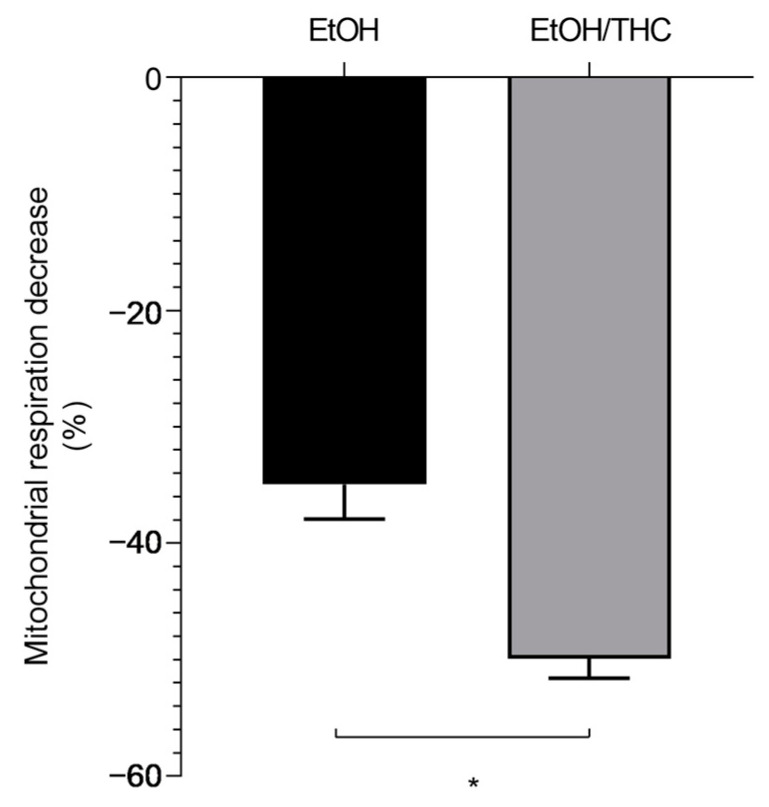
Concomitant THC and EtOH effects are more deleterious on 12-week glycolytic muscle than EtOH alone. These values were obtained for 2.1 × 10^−5^ M EtOH, and 0.2 × 10^−3^ M THC/EtOH. Values are means ± SEM. * *p* < 0.05 EtOH: ethanol. THC: tetrahydrocannabinoid. n = 4 for 12 weeks; n = 3 for 49 weeks.

Aiming to investigate whether the association of EtOH with THC might be more deleterious than EtOH alone, we compared both situations. Interestingly, young glycolytic muscles are more prone to mitochondrial impairment than middle-aged muscles. Thus, at 12 weeks, concomitant THC- and EtOH-induced decreases in mitochondrial respiration were more severe than that observed with EtOH alone (−49.92 ± 1.69 vs. −34.97 ± 2.97%, respectively, *p* < 0.05 (Figure 4)).

**Figure 5:**
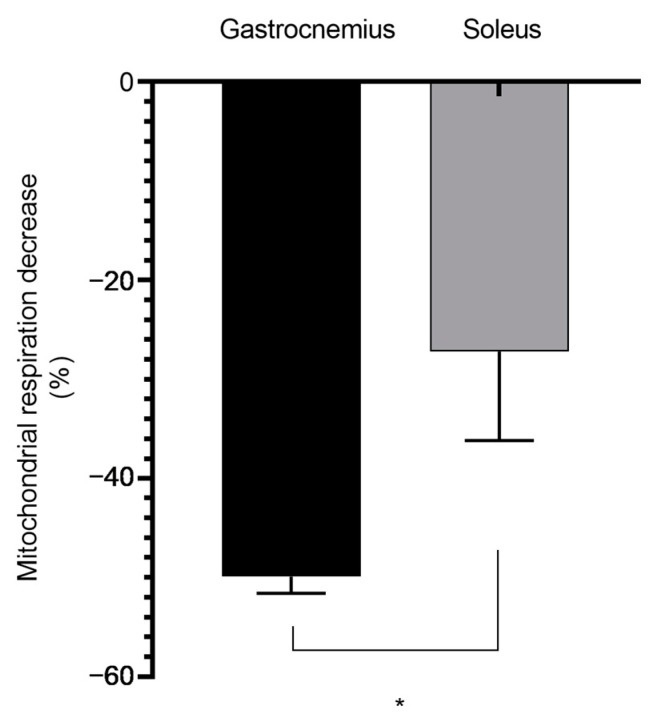
Protective effect of the oxidative phenotype of young muscles against EtOH associated with THC-related deleterious effects. Comparison of gastrocnemius and soleus after injection of 0.2 × 10^−3^ M THC/EtOH at 12 weeks. Values are means ± SEM. * *p* < 0.05. n = 4 for 12 weeks; n = 3 for 49 weeks.

Further, to determine the potential effect of muscle metabolic phenotype, we compared the glycolytic gastrocnemius and the oxidative soleus. Interestingly, oxidative young muscles appeared less severely impacted by EtOH associated with THC than glycolytic muscles. Thus, at 12 weeks, the maximal decrease in mitochondrial respiration was −49.92 ± 1.69 vs. −27.22 ± 8.96% for gastrocnemius and soleus, respectively, at *p* < 0.05 (Figure 5).

At 49 weeks, the maximal mitochondrial respiration decrease was similar in the gastrocnemius and soleus (−31.79 ± 2.36 vs. −28.32 ± 7.32%). Concerning EtOH alone, both young and middle-aged muscles were similarly impaired whatever their metabolic phenotype. 

## 4. Discussion

The main results of this study are as follows: both EtOH alone and EtOH associated with THC significantly impair skeletal muscle mitochondrial respiration, and THC aggravates the deleterious effects of EtOH in young glycolytic muscle. Further, age and metabolic phenotypes modulate these deleterious effects. Thus, glycolytic young muscles appeared more prone to impairments than oxidative muscles. 

### 4.1. THC in Association with EtOH and EtOH Alone Significantly Decreased Skeletal Muscle Mitochondrial Respiration

Both THC + EtOH and EtOH alone demonstrated deleterious effects on muscles’ mitochondrial respiration. This was not unexpected since previous data supported direct deleterious effects of THC or EtOH on several organs including the brain and the heart [17,18,52,53]. Further, THC can be observed in skeletal muscles since it is quickly absorbed after marijuana smoking [54,55,56,57]. 

The mitochondrial respiration decrease was dose-dependent and high doses were needed to alter the skeletal muscles as compared to the doses altering isolated cardiac or brain mitochondria [17,20]. Nevertheless, caution applies when comparing doses in two different situations. Indeed, isolated mitochondria (heart and brain) might show a greater sensitivity to damage than mitochondria preserved by their environment (skeletal muscles fibers) [47,48,49,50]. Additionally, it is well known that both the brain and heart are highly vulnerable to direct damage. 

Further, although studying muscle fibers appeared adequate, more integrated effects of THC and EtOH deserve to be studied. Indeed, small changes in muscles might impair behavior and locomotion. In healthy, physically active young men, strength was not modified after chronic use of marijuana, but anaerobic fatigue tended to be greater in cannabis users as compared to non-users [58]. Accordingly, in a nonhuman primate model, prenatal THC exposure had few effects on fetal and infant muscle development, but even if THC exposition during the gastrula phase exhibited small changes in muscle morphology, it nevertheless may reduce zebrafish activity [59,60].

### 4.2. Metabolic Phenotype and Age Modulate THC- and/or EtOH-Induced Deleterious Effects

Concerning the metabolic characteristics of muscles, the oxidative phenotype is characterized by a greater number of mitochondria per muscle unit (about two-fold greater) and by an enhanced oxidative phosphorylation, as compared to glycolytic muscles [61,62,63]. Accordingly, at 12 weeks, we observed greater baseline soleus mitochondrial respiration as compared to the gastrocnemius. This is consistent with previous data, with both muscles being classified as mainly oxidative or glycolytic, respectively [26,61,62,63,64].

Interestingly, the decrease in mitochondrial respiration was significantly reduced in young soleus as compared to young gastrocnemius. This suggests that an oxidative metabolic phenotype might reduce THC- and EtOH-induced deleterious effects. To the best of our knowledge, this is the first time these data have been reported.

These results are in line with previous reports demonstrating that induction of mitochondrial biogenesis protects against apoptosis in L6 myoblasts [65]. Further, oxidative muscles are more resistant to ischemia–reperfusion, especially when considering mitochondrial respiration, than glycolytic muscles. Thus, soleus and extensor digitorum longus (EDL) muscles, representative of oxidative and glycolytic muscle fiber types, were used to investigate the deleterious effects of lower-limb IR. IR-induced damaged mitochondria, myofibrils and contractile dysfunction were reduced in the *soleus* as compared to EDL. Accordingly, IR-induced skeletal muscle rhabdomyolysis is a fiber type-specific phenomenon, modulated by mitochondria reserves [24,25,26,66]. Further supporting our results, Decker et al. recently demonstrated that cigarette smoke condensate-induced impairment in mitochondrial respiration was enhanced in the gastrocnemius as compared to the soleus [64].

Confirming this hypothesis, in middle-aged muscles where no difference was observed in terms of mitochondrial respiration, the THC- and EtOH-related decrease in mitochondrial respiration was similar in both gastrocnemius and soleus muscles. 

Analyzing the effects of EtOH alone, middle-aged oxidative muscles tend to be protected compared to the middle-aged gastrocnemius. The difference did not reach statistical significance, but this might suggest that an oxidative metabolic phenotype also protects against ethanol-related mitochondrial dysfunction.

Concerning age, the decrease in gastrocnemius mitochondrial respiration was greater in young than in middle-aged muscle after THC exposure. After EtOH exposure, soleus alteration in muscles was also greater at 12 weeks than at 49 weeks. These data provide evidence that young skeletal muscle might be less resistant to both THC- and EtOH-induced deleterious effects. Usually, older muscles are more sensitive to injuries, but this is mainly true when considering very old muscles of 80–90 weeks. In this study, we analyzed middle-aged muscle that might be at their apogee in terms of defense abilities against damage [67].

### 4.3. Relative Contributions of THC and Ethanol on Skeletal Muscles Mitochondrial Respiration Impairments

Since EtOH is often concomitantly used with THC, analyzing such an association appeared pertinent. When subtracting the effect of EtOH alone from the effect of THC dissolved in EtOH, we approached the respective actions of the two compounds on the skeletal muscles. Interestingly, EtOH was more deleterious, inducing a greater decrease in mitochondrial respiration than THC. This was observed in both glycolytic and oxidative muscles, whatever the age. In fact, alcohols are known to reduce mitochondrial respiration and methanol and EtOH decreased mitochondrial oxidative phosphorylation with an IC50 of 8.3% (*v*/*v*) and 4.6% [68]. Accordingly, mitochondria are altered after acute alcohol exposure [38,69]. Thus, alcohol-related myopathy rely also on mitochondrial impairments with impaired cellular respiration in myotubes and muscles [70,71]. 

## 5. Conclusions

In conclusion, both EtOH alone and EtOH associated with THC significantly impair skeletal muscle mitochondrial respiration. Further, concomitant THC can increase the deleterious effects of EtOH and glycolytic young muscles appeared more prone to impairments than oxidative muscles.

These data support the need for caution when considering using THC for neuromuscular disease and/or pain therapies, or recreational use. Indeed, humans often use both drugs concomitantly [72] and besides addiction, cognition alteration and/or cardio-vascular diseases, they might suffer from further muscular impairments. In addition, deleterious effects might also be enhanced by common other risk factors such as a high-fat diet, which exacerbate the negative effects of alcohol on skeletal muscle mitochondrial health [73]. 

## Data Availability

Data contained within the article.
